# The Evolutionary Constraints on Angiosperm Chloroplast Adaptation

**DOI:** 10.1093/gbe/evad101

**Published:** 2023-06-03

**Authors:** Elizabeth H J Robbins, Steven Kelly

**Affiliations:** Department of Biology, University of Oxford, Oxford, United Kingdom; Department of Biology, University of Oxford, Oxford, United Kingdom

**Keywords:** chloroplast, evolvability, adaptive evolution, evolutionary constraint, phylogenomics

## Abstract

The chloroplast (plastid) arose via the endosymbiosis of a photosynthetic cyanobacterium by a nonphotosynthetic eukaryotic cell ∼1.5 billion years ago. Although the plastid underwent rapid evolution by genome reduction, its rate of molecular evolution is low and its genome organization is highly conserved. Here, we investigate the factors that have constrained the rate of molecular evolution of protein-coding genes in the plastid genome. Through phylogenomic analysis of 773 angiosperm plastid genomes, we show that there is substantial variation in the rate of molecular evolution between genes. We demonstrate that the distance of a plastid gene from the likely origin of replication influences the rate at which it has evolved, consistent with time and distance-dependent nucleotide mutation gradients. In addition, we show that the amino acid composition of a gene product constraints its substitution tolerance, limiting its mutation landscape and its corresponding rate of molecular evolution. Finally, we demonstrate that the mRNA abundance of a gene is a key factor in determining its rate of molecular evolution, suggesting an interaction between transcription and DNA repair in the plastid. Collectively, we show that the location, the composition, and the expression of a plastid gene can account for >50% of the variation in its rate of molecular evolution. Thus, these three factors have exerted a substantial limitation on the capacity for adaptive evolution in plastid-encoded genes and ultimately constrained the evolvability of the chloroplast.

**Significance**

The chloroplast genome in plants is highly conserved with little variation in gene order or gene content. However, there is a surprising variation in the rate of evolution between genes. We found that just three factors—the location, the composition, and the expression of a gene—could explain ∼50% of the variation in gene evolutionary rate between plastid genes. These three factors impose a substantial limitation on the capacity for adaptive evolution of plastid genes and ultimately constrain the evolvability of the chloroplast.

## Introduction

The chloroplast (plastid) is a membrane-bound organelle that arose from the endosymbiosis of a photosynthetic cyanobacterium by a nonphotosynthetic eukaryotic cell ∼1.5 billion years ago (Schwartz and Dayhoff 1978; Martin and Kowallik 1999; Hedges et al. 2004). Early in the transition from cyanobacterium to organelle, the plastid underwent a substantial genome reduction whereby thousands of genes were either lost or transferred to the nucleus (Blanchard and Schmidt 1995; Martin et al. 2002; Kelly 2021). As a consequence, modern-day plastid genomes in plants contain fewer than 5% of the genes found in their free-living bacterial relatives, with the majority of plant plastids containing only ∼80 protein-coding genes (Palmer 1985; Timmis et al. 2004). As a result, despite comprising over 80% of the protein (Makino and Osmond 1991; Li et al. 2017) and 80% of the mRNA (Forsythe et al. 2022) in a plant cell, the combined forces of gene loss and endosymbiotic gene transfer mean that plastids typically contain fewer than 0.5% of the genes found in a typical plant cell.

Although the plastid underwent rapid evolution by genome reduction, the rate of molecular evolution of the plastid-encoded genes has been markedly slow and the organization in the plastid chromosome has been highly conserved. Comparisons between plant species have revealed that the plastid genome has a mutation rate that is an order of magnitude lower than that of the nuclear genome (Wolfe et al. 1987; Smith 2015). The finding that the mutation rate of the plastid was low in comparison to the nuclear genome was initially unexpected given that the plastid genome is predominantly uniparentally inherited (Birky 1995; Mogensen 1996), where an absence of sexual recombination is thought to lead to an accumulation of deleterious mutations in a phenomenon known as Muller's ratchet (Muller 1964). Accordingly, it was proposed that gene conversion in the presence of a high genome copy number per plastid (∼900 per plastid; Bendich 1987) acts to counteract the effect of Muller's ratchet to prevent accumulation of mutations (Birky and Walsh 1992; Khakhlova and Bock 2006). However, it is unclear whether other factors intrinsic to the plastid genome itself also act to limit the rate of molecular evolution of plastid genes.

One factor that could differentially impact the rate of molecular evolution of plastid-encoded genes is their position in the chromosome relative to the point of replication initiation. Most plant species contain a plastid genome that has a quadripartite structure (Palmer 1985; Daniell et al. 2016) composed of two single-copy regions (the large single-copy region and the small single-copy region) separated by two inverted repeat regions. Although multiple replicative mechanisms have been hypothesized (Kolodner and Tewari 1975; Takeda et al. 1992; Kunnimalaiyaan and Nielsen 1997; Oldenburg and Bendich 2004, 2016), the origin(s) of replication have been repeatedly proposed to be located in the inverted repeat regions, and both the replication mechanism and position relative to these origins are thought to impact the evolution of the nucleotide sequence along the plastid genome (Wolfe et al. 1987; Smith 2015). For example, adenine to guanine deamination gradients disseminate away from the inverted repeat regions, and mutation rates in the single-copy regions are much higher than in the inverted repeats (Wolfe et al. 1987; Krishnan and Rao 2009). The formation of the deamination gradients is thought to be attributable to the differential length of time in which the regions of the plastid genome are single stranded during DNA replication, and the difference in mutation rate between the single-copy and inverted repeat regions is thought to be due to enhanced frequency of homologous recombination-mediated repair in the inverted repeat (Khakhlova and Bock 2006). Other context and location-dependent effects on mutation in the plastid genome have been described which also alter the rate and type of mutations that occur along the plastid chromosome (Wolfe et al. 1992; Kelchner 2000; Jansen et al. 2007; Zhu et al. 2016; Niu et al. 2017). However, it is unknown whether these position-dependent effects have influenced the rate of molecular evolution of plastid gene sequences, or how these phenomena interact with other factors to modulate the rate of molecular evolution of plastid-encoded genes.

Here, we present an analysis of the molecular evolution of the single-copy plastid-encoded protein-coding genes from 773 species of angiosperms. We show that there is substantial variation in the rate of molecular evolution between genes, with genes whose products function in energy production having evolved more slowly than those whose products function in information processing (transcription and translation). We then perform an analysis of multiple factors that could contribute to the variation in the rate of molecular evolution between genes, including gene position, amino acid composition, nucleotide composition, levels of gene expression, transcript biosynthesis cost, and transcript translational efficiency. In doing so, we reveal that the level of mRNA abundance of a gene, its position relative to the inverted repeat region, and its amino acid composition each significantly constrain the rate of evolution of plastid-encoded genes. Thus, the combined action of compositional factors and production requirements impose a severe constraint on the molecular adaptation of the chloroplast.

## Results

### There Is a Large Variation in Rate of Molecular Evolution Between Plastid-Located Genes

To understand the factors that have influenced the rate of molecular evolution of plastid-encoded genes, a data set of fully sequenced plastid genomes was compiled. To do this, the full set of sequenced plastid genomes was downloaded from the National Center for Biotechnology and Information (NCBI) (Wolfsberg et al. 2001) and subjected to filtration to remove genomes that did not contain a full set of the genes typically found in angiosperms. This resulted in a data set comprising complete plastid genomes from 773 species of angiosperms, each containing 69 single-copy protein-coding genes that are ubiquitously present and in all species. Gene trees were inferred from nucleotide multiple sequence alignments for all ubiquitously conserved genes (see Materials and Methods), and the phylogenetic trees were used to evaluate and compare the gene-wide rates of synonymous (*d*_S_) and nonsynonymous substitution (*d*_N_). The synonymous substitution rate is computed from mutations that do not alter the encoded protein and thus informs the background substitution rate to which the gene is exposed. In contrast, the nonsynonymous substitution rate is computed from mutations that alter the encoded protein sequence and shows the rate at which the protein is evolving. Moreover, the ratio of the rate of nonsynonymous substitution to synonymous substitution (*d*_N_/*d*_S_) indicates the strength of selection to which a gene is subject, whereby genes with smaller values encode proteins subject to greater purifying selection due to increased functional constraint.

The total length of each gene tree, that is, the sum of all the branch lengths in the gene tree, was used as the estimate for the rate of synonymous substitution and the rate of nonsynonymous substitution for each gene, respectively. This measure accounts for differences in alignment length between genes because the branch lengths in the tree are estimates of the number of substitutions per sequence site (supplementary fig. S1, Supplementary Material online). This measure is also not affected by species sampling, variation in model of sequence evolution, or variation in topology of the underlying tree, as each of these factors was held constant in all analyses that were conducted. Specifically, all 773 plant species are in each gene tree, and each gene is inferred from a strictly single-copy gene in each species. Furthermore, the topology of each gene tree was constrained to the consensus topology inferred from a concatenation of all genes (see Materials and Methods), and the model of codon evolution was held constant across all individual tree inferences using parameters that allow for comparison of branch lengths between trees. Thus, this analysis removes any potential phylogenetic bias that could occur from variation in these factors.

**Fig. 1.**
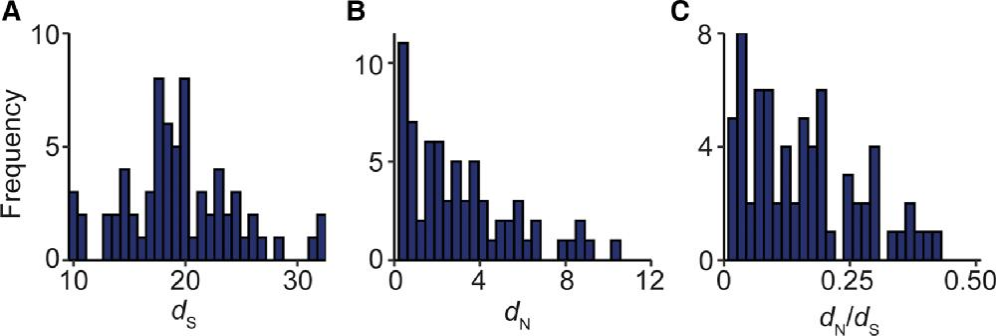
The rates of molecular evolution for 69 single-copy protein-coding plastid genes. (*A*–*C*) Frequency distributions of the rates of synonymous substitution (*d*_S_), nonsynonymous substitution (*d*_N_), and strength of selection (*d*_N_/*d*_S_), respectively. *d*_S_ has the units of number of synonymous substitutions per synonymous sequence site and *d*_N_ has the units of the number of nonsynonymous substitutions per nonsynonymous sequence site.

Comparison of the rates of synonymous substitution between genes revealed that there was a 3-fold difference between the genes with the fastest and slowest background substitution rate, corresponding to *ndhF* and *psbF*, respectively (fig. 1*A* and supplementary table S1, Supplementary Material online). In contrast, the rate of nonsynonymous substitution varied much more substantially with a 47-fold difference between genes with the fastest and slowest evolving encoded proteins, *rpl22* and *atpH*, respectively (fig. 1*B* and supplementary table S1, Supplementary Material online). Moreover, although all gene-wide values of *d*_N_/*d*_S_ were small (<0.5), there was still a 23-fold difference between genes subject to the weakest and strongest purifying selection, *matK* and *psaC*, respectively (fig. 1*C* and supplementary table S1, Supplementary Material online). Thus, even though the plastid genome has evolved slowly in comparison with the nuclear genome (Wolfe et al. 1987; Smith 2015), there is still substantial variation in rate of background substitution, rate of protein evolution, and functional constraint between plastid-encoded genes.

### Plastid Genes With Functions in Energy Production Have Evolved Slower and Are More Constrained Than Genes That Function in Information Processing

**Fig. 2.**
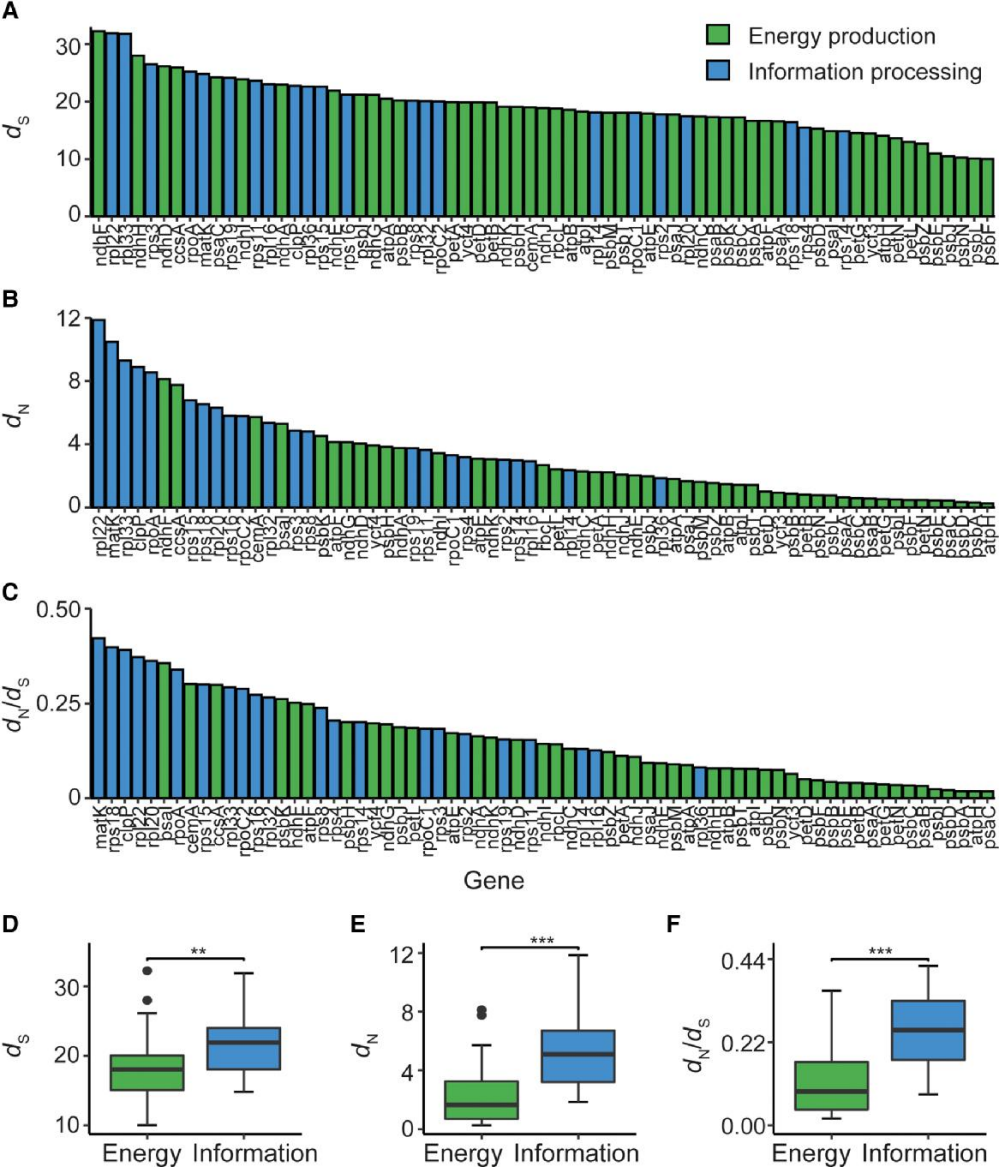
Relationship between gene function with rate of molecular evolution and functional constraint. (*A*–*C*) Bar graphs showing *d*_S_ (*A*), *d*_N_ (*B*), and *d*_N_/*d*_S_ (*C*) for individual genes in order of descending value from left to right. Genes are colored by their high-level function: green, energy production; blue, information processing. The energy production category includes genes that function in or are associated with photosystem I, photosystem II, cytochrome b_6_f complex, ATP-synthase, NAD(P)H dehydrogenase-like complex, or rubisco. The information processing category includes genes involved in mRNA maturation in addition to genes that encode subunits of the 70S ribosome, the plastid-encoded RNA polymerase, and the plastid protease system. (*D*–*F*) The difference in *d*_S_ (*D*), *d*_N_ (*E*), and *d*_N_/*d*_S_ (*F*) between genes involved in energy production (green, *n* = 47) versus information processing (blue, *n* = 22), respectively. Statistical significance was assessed using a Welch's *t*-test (*P* < 0.01, indicated by **) for *d*_S_ and a Wilcoxon test for *d*_N_ and *d*_N_/*d*_S_ (both *P* < 0.001, indicated by ***).

Inspection of the rank order of the rate of synonymous substitution, rate of nonsynonymous substitution, and strength of purifying selection (fig. 2*A*–*C*) suggested that genes involved in energy production (i.e., genes whose products function in photosystem I, II, cytochrome b_6_f complex, ATP-synthase, and the NAD(P)H dehydrogenase-like complex or CO_2_ fixation) had lower rates of molecular evolution and were subject to greater purifying selection than those involved in information processing (i.e., genes that function in transcription, mRNA maturation, translation, or protein degradation). Comparison of these two groups revealed that genes with functions in energy production had evolved slower and were subject to greater purifying selection than those with functions in information processing, with a mean fold difference of 1.2 in the rate of synonymous substitution (Welch's *t*-test, *P* < 0.01) and median fold differences of 3.1 and 2.8 in the rate of nonsynonymous substitution and the ratio of nonsynonymous to synonymous substitution (Wilcoxon tests, both *P* < 0.001) (fig. 2*D*–*F*). Within each category, there was little to no difference in the evolutionary rate or functional constraint of genes between multiprotein complexes (supplementary fig. S2, Supplementary Material online). Thus, by all measures considered here, genes with functions in energy production have evolved slower and are more constrained than genes that function in information processing.

### The Location of a Gene in the Plastid Genome Has Influenced Its Rate of Molecular Evolution

**Fig. 3.**
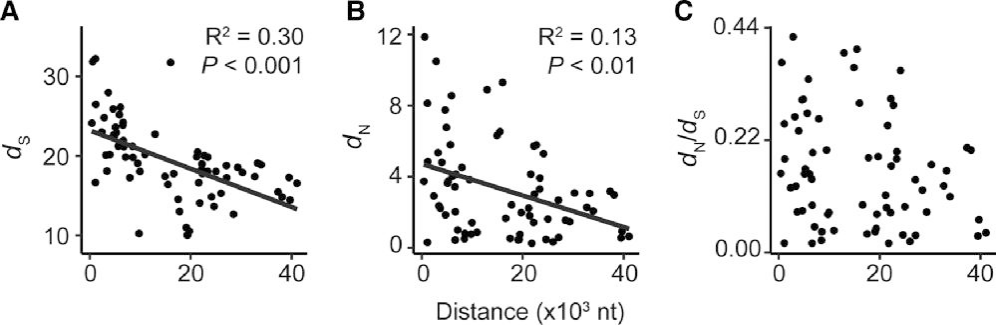
The relationship between the average distance of a gene to the closest inverted repeat border and the rate of molecular evolution of 69 single-copy plastid-encoded genes. Scatter plots showing the average distance of each gene's midpoint to the closest inverted repeat border measured in nucleotides (nt) versus the rate of synonymous substitution (*A*), nonsynonymous substitution (*B*), and functional constraint (*C*). Where appropriate, linear models are shown in black with their associated *R*^2^ and *P* values.

As noted in the introduction, the rate and type of mutation along the plastid genome is nonuniform. Therefore, it is likely that the position of a gene in the plastid genome could influence its rate of molecular evolution. Given that all the genes analyzed here are in the single-copy regions of the plastid genome, gene position was assessed by calculating the average distance from each gene's midpoint to the closest border of an inverted repeat region in all 773 species. This distance was then compared with the rate of synonymous substitution, nonsynonymous substitution, and functional constraint of each gene. This revealed that the further away a gene is located from the inverted repeat, the lower its rate of synonymous substitution (fig. 3*A*). In total, distance to the inverted repeat border could explain ~30% of the variance the rate of synonymous substitution between genes (*R*^2^ = 0.30, *P* < 0.001). A similar effect was observed for the rate of nonsynonymous substitution (*R*^2^ = 0.13, *P* < 0.01) (fig. 3*B*). In contrast, a significant association was not observed between average distance to the inverted repeat border and the strength of selection acting on the encoded protein (fig. 3*C*). Similar relationships were observed if the rates of molecular evolution and strength of selection of individual genes were aggregated at the level of operons (supplementary fig. S3, Supplementary Material online). Thus, all locations in the plastid genome are not equal, and the rate at which a plastid gene has evolved during the radiation of the angiosperms is dependent on how distant that gene is positioned from the inverted repeat in the plastid genome.

### Physicochemical Constraints on Amino Acid Substitution Have Modulated the Strength of Purifying Selection Acting on Plastid-Encoded Genes

**Fig. 4.**
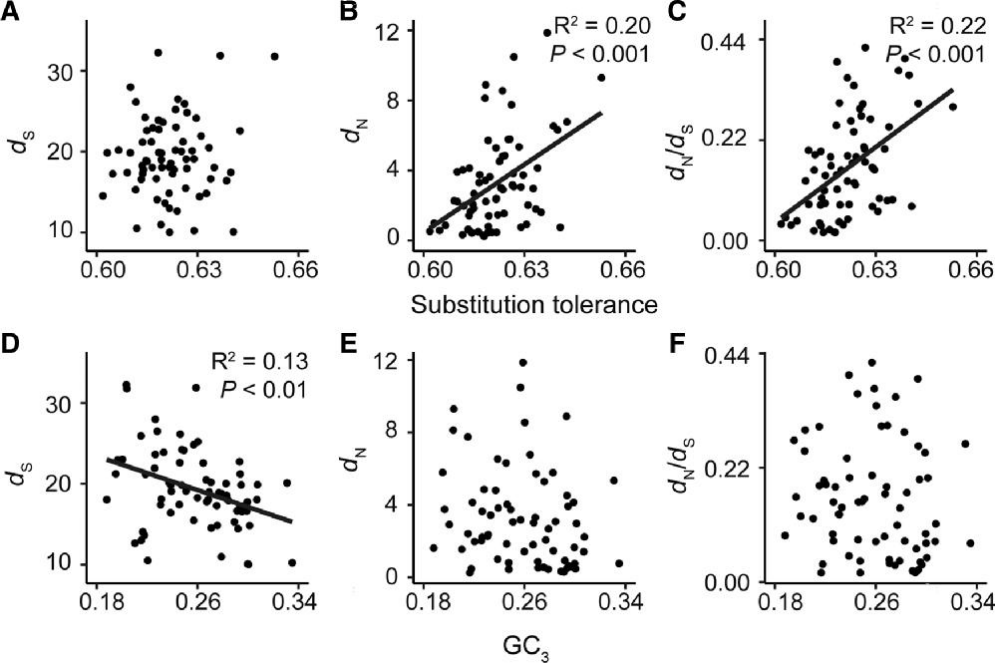
The effect of gene composition on the rate of molecular evolution for plastid-encoded genes. The nucleotide composition of a gene was determined by analyzing the GC content at the third codon position (GC_3_). Meanwhile, amino acid composition of the encoded protein was summarized in a substitution tolerance score based on the BLOSUM62 matrix, where a lower value corresponds to proteins with a composition less tolerant to substitution. (*A*–*C*) Scatter plots showing protein substitution tolerance scores versus the rate of synonymous substitution, rate of nonsynonymous substitution, and the ratio of nonsynonymous substitution to synonymous substitution, respectively. (*D*–*F*) Scatter plots showing GC_3_ versus the rate of synonymous substitution, rate of nonsynonymous substitution, and the ratio of nonsynonymous substitution to synonymous substitution, respectively. Where appropriate, linear models are shown in black with their associated *R*^2^ and *P* values.

Given that the functional constraint of a protein was independent of any genome position effects, we sought to determine whether any component of this constraint was attributable to compositional differences between the encoded protein sequences. Such a compositional effect may arise from the biochemical differences and similarities between amino acid monomers found in a given protein sequence. This is because amino acids are not all equally dissimilar to each other, and many share similarities in their size, charge, and reactivity of their side chains. The extent of similarity between amino acids varies such that each different amino acid monomer has a different tolerance to substitution in protein sequences (Sneath 1966; Epstein 1967; Grantham 1974; Henikoff and Henikoff 1992). Accordingly, variation in constraint on the rate of nonsynonymous substitution could arise simply from compositional differences between genes. A substitution tolerance can be effectively described using substitution frequency data summarized in the BLOSUM62 amino acid substitution matrix (Henikoff and Henikoff 1992). We hypothesized that proteins containing a higher proportion of amino acids that were less tolerant to substitution would have slower protein sequence evolution and would be subject to stronger purifying selection because mutations would be more likely to be deleterious to the encoded protein sequence. To investigate this, a substitution tolerance score was calculated for each gene (see Materials and Methods) and compared with the rate of synonymous substitution, rate of nonsynonymous substitution, and strength of purifying selection of that gene. Although there was no association between the substitution tolerance of an encoded protein with the rate of synonymous substitution (fig. 4*A*), there were significant associations between substitution tolerance and the rate of nonsynonymous substitution (*R*^2^ = 0.20, *P* < 0.001) (fig. 4*B*) and the strength of purifying selection acting on the gene (*R*^2^ = 0.22, *P* < 0.01) (fig. 4*C*). Here, genes with lower substitution tolerance scores had lower rates of nonsynonymous substitution and were subject to stronger purifying selection. Thus, all sequences are not equal, and the rate at which a plastid gene has evolved during the radiation of the angiosperms is dependent on the amino acid composition of that gene.

### The Intrinsic Nucleotide Composition Has Only Weakly Influenced the Rate of Background Substitution

Given that the composition of the encoded amino acid sequence has contributed significantly to the rate at which the underlying molecular sequence of a gene has evolved, it was next determined whether similar composition-based constraints were manifest at the level of the nucleotide sequence. There is some precedent for hypothesizing that this might occur, as genes encoded in the inverted repeat regions have higher guanine–cytosine (GC) contents and have evolved more slowly than genes located in the single-copy regions (Li et al. 2016), and accordingly similar effects may be apparent at the level of an individual gene. To prevent redetecting differences in amino acid composition at the nucleotide level, only the GC content of the third codon position (GC_3_) was investigated, excluding amino acids that have no degeneracy at this position. Comparison of the GC_3_ content of a gene to its rate of synonymous substitution, nonsynonymous substitution, and strength of purifying selection revealed that the GC_3_ could explain ∼13% of the variance in the rate of synonymous substitution (*R*^2^ = 0.013, *P* < 0.01), where genes with higher GC_3_ contents have evolved more slowly (fig. 4*D*). Meanwhile, no significant relationships were identified between GC_3_ and the rate of nonsynonymous substitution and strength of purifying selection (fig. 4*E* and *F*). This latter result was not surprising given that there was no relationship between GC_3_ and the GC content of the first two codon positions, that is, the positions that are major determinants of residue identity (supplementary fig. S4, Supplementary Material online). Thus, whereas genes that have higher GC_3_ contents had slower evolving nucleotide sequences, this interaction is weak and GC_3_ has not had an impact on the rate of protein evolution or strength of protein functional constraint.

### mRNA Abundance Is a Determinant of Plastid Gene Evolutionary Rate

**Fig. 5.**
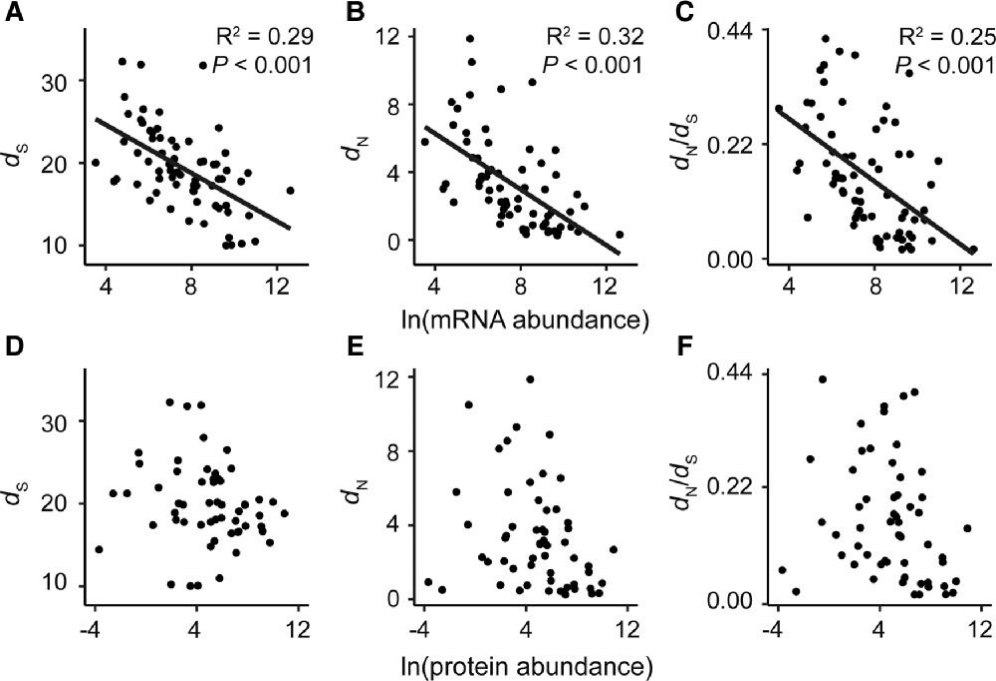
Relationship between the rate of molecular evolution and gene expression for plastid-encoded protein-coding genes. (*A*–*C*) Scatter plots of mRNA abundance (data from Forsythe et al. 2022) versus the rate of synonymous substitution, rate of nonsynonymous substitution, and the ratio of nonsynonymous substitution to synonymous substitution, respectively. Linear regressions are shown as black lines with their associated *R*^2^ and *P* values. (*D*–*F*) Scatter plots of protein abundance (data from Wang et al. 2012) versus rate of synonymous substitution, rate of nonsynonymous substitution, and the ratio of nonsynonymous substitution to synonymous substitution, respectively.

High gene expression has been previously linked to low rates of molecular evolution in multiple systems (Pál et al. 2001; Subramanian and Kumar 2004; Wright et al. 2004; Seward and Kelly 2018). To investigate whether this phenomenon was also true of genes encoded in the plastid genome, the rate of molecular evolution and strength of purifying selection of a plastid gene was compared with both the mRNA and protein abundance of that gene. In agreement with previous analysis in other systems, genes with high mRNA abundance had low levels of molecular evolution and vice versa (fig. 5). Specifically, variance in mRNA abundance could explain 29% of the variation in the rate of synonymous substitution (*R*^2^ = 0.29, *P* < 0.001) (fig. 5*A*), 32% of variation in the rate of nonsynonymous substitution (*R*^2^ = 0.32, *P* < 0.001) (fig. 5*B*), and 25% of variation in the strength of purifying selection (*R*^2^ = 0.25, *P* < 0.001) (fig. 5*C*), respectively. In contrast, there was no significant relationship between protein abundance and rate of molecular evolution or strength of purifying selection (fig. 5*D*–*F*). This suggests that the constraint on the evolutionary rate is mediated via an interaction with transcription rather than through the requirement for protein production. Furthermore, previously detected interactions between mRNA abundance and gene evolutionary rate have been manifested in part through selection acting on transcript biosynthesis cost (the number and type of atoms contained within the transcript and the number of high-energy phosphate bonds required for biosynthesis) and transcript translation efficiency (a function of the number of tRNAs that can translate that codon encoded in the plastid genome) (Seward and Kelly 2016, 2018). However, there was no evidence for an interaction between these two factors and variation in the rate of molecular evolution or the strength of purifying selection of genes encoded in the plastid genome (supplementary figs. S5 and S6, Supplementary Material online). Thus, the extent to which a transcript accumulates in the chloroplast has constrained the evolutionary rate of its corresponding gene, and this is independent of selection acting to preserve the biosynthetic cost, or translational efficiency of the coding sequence, or the abundance of the encoded protein.

### Covariate Models Can Explain >50% of the Variance in the Rate of Molecular Evolution of Plastid-Encoded Genes

**Fig. 6.**
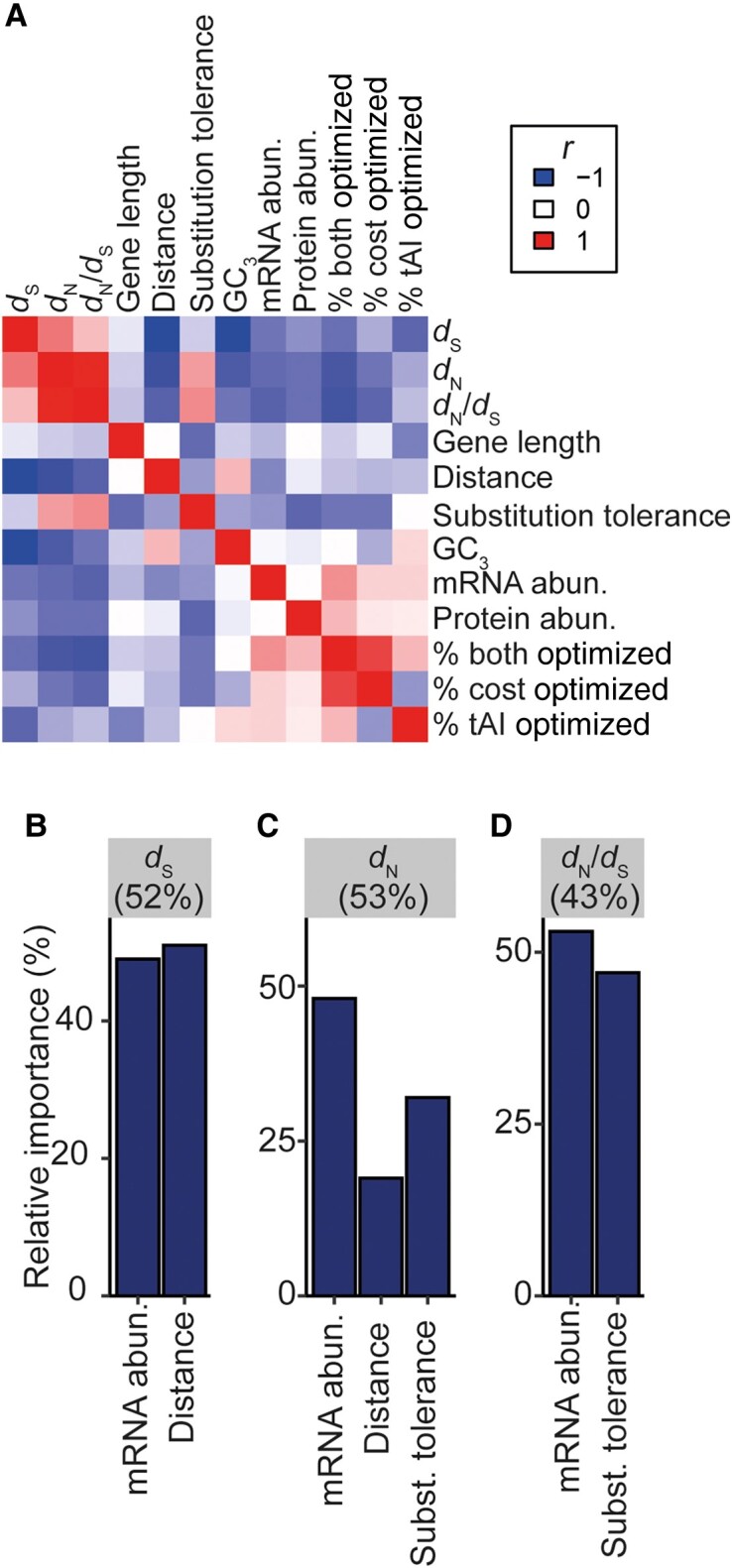
Relative importance of covariates in the best linear models identified to explain the rates of molecular evolution. (*A*) A correlation heatmap of all continuous variables used in this study. Red, Pearson correlation coefficient (*r*) = 1; white, *r* = 0; blue, *r* = −1. (*B*–*D*) The relative importance (given as a percentage) of mRNA abundance, gene distance to the inverted repeat, and protein substitution tolerance in explaining 52%, 53%, and 43% of the variance in the rate of synonymous substitution, rate of nonsynonymous substitution, and the purifying selection, respectively.

Given that several factors were identified that could each explain a significant component of variation in the rate of molecular evolution of plastid genes in isolation, we next sought to identify the cumulative variance attributable to all factors while accounting for interdependencies (fig. 6*A*) which may deflate the explained variance observed when single variables are considered. To do this, regression tests were conducted for all possible subsets of the set of independent variables described above. This revealed that gene position and mRNA abundance could explain 52% of the variation in the rate of synonymous substitution (figs. 6*B* and supplementary fig. S7, Supplementary Material online). Relative importance metrics showed gene position and mRNA abundance were similarly important covariates, contributing to 51% and 49% of the total variance explained, respectively (fig. 6*B*). A similar proportion of variance in the rate of nonsynonymous substitution could be explained by the same factors above with the addition of the protein's substitution tolerance (figs. 6*C*, 53% variance explained, and supplementary fig. S8, Supplementary Material online). Specifically, mRNA was the most important covariate (48% variance explained), followed by protein substitution tolerance (32% variance explained) and then gene position with respect to the inverted repeat border (19% variance explained) (fig. 6*C*). Although protein sequence constraint is independent of gene position, mRNA abundance and protein substitution tolerance together explained 43% of the variation in purifying selection between genes, contributing 53% and 47% of the explained variance, respectively (figs. 6*D* and S9, Supplementary Material online). Thus, in total, >50% of the variance in the rate of molecular evolution and >40% of the variance in strength of purifying selection are attributable to compositional factors and production requirements. In all cases, the weak associations between GC_3_ content and the rates of molecular evolution and strength of purifying selection (fig. 6) were rendered insignificant when other factors above were accounted for.

To ensure model space was effectively explored, random forests were also run on the complete set of factors to determine if there were alternative models that could explain the variance in the rate of molecular evolution or the strength of purifying selection (supplementary file 1, Supplementary Material online). None of the random forest models outperformed the linear regression analyses shown above, despite including more parameters (supplementary file 1, Supplementary Material online). Thus, mRNA abundance, gene position, and protein tolerance to substitution together account for >50% of the variance in the molecular evolution and >40% of the variance in protein sequence constraint in the plastid genome, and have substantially constrained the capacity for adaptive evolution in the chloroplast genome.

## Discussion

The endosymbiosis of the bacterial progenitor of the chloroplast is a landmark event in the evolution of eukaryotes. It first enabled oxygenic photosynthesis in eukaryotes and helped direct the Earth on a continued trend of decreasing atmospheric CO_2_ and increasing O_2_ over the subsequent ∼1.5 billion years (Holland 2002; Canfield 2005). Following this endosymbiosis, there was a dramatic reduction in the gene content of the plastid genome, such that it now harbors fewer than 5% of the genes found in its free-living bacterial relatives (Palmer 1985; Timmis et al. 2004). These remaining genes have been trapped in the same genome and inherited in the absence of sexual recombination throughout their evolution (Birky 1995; Mogensen 1996). Here, we investigate the factors that have contributed to the variation in evolutionary rate of plastid-encoded genes over the last 125 Myr during the radiation of the angiosperms. We show that the rate of molecular evolution has been governed by factors extrinsic to gene function, specifically the position of the gene in the plastid genome, the substitution tolerance of the encoded protein, and the level of gene expression. Combined, these production requirements and compositional factors explain >50% of the variation in the rates of synonymous and nonsynonymous substitution; moreover, they also explain >40% of the variation in the rate of purifying selection. Thus, the capacity for molecular evolution of a plastid-encoded gene has been determined to a large extent by the location, composition, and expression level of that gene.

The finding that genes which are positioned further from the inverted repeat border, and hence the likely origin of replication, are evolving more slowly than genes that are closer has two of important implications. First, this finding is compatible with models of chloroplast DNA replication where replication initiates at origins located in the inverted repeat and where DNA closer to the inverted repeat border spends more time in a single-stranded state than DNA that is more distant to the origin. This is because single-stranded DNA is more susceptible to base deamination which can result in increased likelihood of mutation and therefore elevated rates of evolution (Lindahl 1993; Krishnan et al. 2004). Thus, if DNA closer to the origin of replication spends more time single stranded during replication, this could lead to a positional effect where genes closer to the inverted repeat have elevated rates of molecular evolution. One model of plastid genome replication that satisfies both these criteria is the bidirectional dual displacement loop mechanism (Kolodner and Tewari 1975). However, there is increasing evidence that plastid genome replication proceeds via a recombination-dependent mechanism (Oldenburg and Bendich 2004, 2016), and although these mechanisms have also been proposed to initiate in the inverted repeat, it is difficult to conceptualize how DNA would spend differential time single stranded in these models. Therefore, our findings are consistent with the bidirectional dual displacement loop mechanism and lend further support to analyses of intergenic regions and the third codon position that indicate the widespread operation of this form of plastid DNA replication in angiosperms (Krishnan and Rao 2009). Second, the fact that origin-proximal genes are evolving faster than origin-distal genes means that all locations in the plastid genome are not equal in terms of their capacity to generate genetic diversity. Accordingly, the genes located further from the inverted repeat have had the capability to explore more sequence space and have generated more diversity on which natural selection can act, during angiosperm evolution. Thus, the position of a gene and the structure of the plastid genome have constrained the evolvability of adaptive changes in the chloroplast.

The 20 amino acids that are used to build proteins vary in the physical and chemical properties of their side chains: some are hydrophobic, some are charged, some are large, and some are small. Consequently, a mutation that results in the substitution of one amino acid for another with similar physicochemical properties is more likely to have a smaller impact on the structure and function of a protein than substitution with an amino acid with substantially different properties (Sneath 1966; Epstein 1967; Henikoff and Henikoff 1992; Bohórquez et al. 2017). As there are different numbers of similar amino acids in each physicochemical category, and each amino acid can occupy different physicochemical categories, each amino acid therefore has a different tolerance to substitution. We estimated the substitution tolerance of each protein sequence encoded in the plastid genome and found that the aggregate substitution tolerance of a protein sequence is a major component of variation in its evolutionary rate. This means that the evolvability of novel adaptive phenotypes arising from variation in plastid-encoded genes is limited by the identity of the encoded amino acids in that gene.

In agreement with previous analyses in other systems, we observed that plastid genes with high expression levels had lower rates of molecular evolution (Pál et al. 2001; Subramanian and Kumar 2004; Wright et al. 2004; Seward and Kelly 2018). However, in contrast to previous studies, this phenomenon was not attributable to selection acting to maintain a low transcript biosynthesis cost or a high translational efficiency, nor was it attributable to the requirement for high protein abundance. The lack of association with protein abundance could be due to increased noise in the data as it is an integrated data set, or alternatively, protein abundance is not a good proxy for protein production as protein turnover is not accounted for. Nevertheless, the results presented here raise the question as to what phenomenon could give rise to a strong interaction between mRNA abundance with the rate of molecular evolution that is not attributable to transcript biosynthetic cost, translational efficiency, or protein abundance. One mechanism that could potentially explain this dependency is the presence of transcription-mediated DNA repair, whereby increased transcriptional activity directly enhances DNA repair. This would give rise to a phenomenon whereby the more highly transcribed a gene, the more likely DNA damage is repaired in that gene and the lower its mutation rate. Interestingly, this potential explanation is supported by the presence of a nuclear-encoded, chloroplast-targeted ortholog of mutation frequency decline protein (Mfd), alternatively named transcription-repair coupling factor (Hays 2002). In bacteria, Mfd facilitates DNA repair at stalled RNA polymerase complexes by recruiting the nucleotide excision repair machinery (Selby and Sancar 1993). Additionally, it is proposed to play a role in promoting homologous recombination-mediated repair (Ayora et al. 1996). Although plants lack orthologs of other nucleotide excision repair genes (Hays 2002), they contain orthologs of the recombination-mediated repair factors (Maréchal and Brisson 2010) and recombination-based repair is a primary DNA repair mechanism in the plastid (Cerutti et al. 1995; Khakhlova and Bock 2006; Maréchal and Brisson 2010). Thus, it is possible that Mfd in plants functions to promote recombination-based repair in response to DNA damage-stalled RNA polymerases. This effect would likely arise from Mfd's ability to form R-loops by distorting the DNA around the stalled polymerase (Portman et al. 2021). This, in turn, is known to lead to recruitment of plastid homologous recombination machinery to facilitate DNA repair (Wang et al. 2021). Thus, although it is impossible to prove the existence of transcription-coupled DNA repair from the data presented here, it is consistent with the well-described phenomenon. Furthermore, it will be interesting to determine whether loss of function of Mfd in plants leads to increased mutation in the chloroplast genome in a manner that is consistent with a reduction in transcription-mediated DNA repair and thus can provide a mechanistic explanation for the transcription-dependent constraint on the rate of molecular evolution of plastid genes.

Our finding that the expression, location, and amino acid composition of a gene impose significant constraints on the rate of molecular evolution of plastid-encoded genes is important. It means that factors other than the function of the encoded protein have been important in constraining the evolvability of plastid genes. In this context, it is noteworthy that the constraints identified here help provide a mechanistic explanation for the strong phylogenetic constraints that have limited the adaptation of rubisco enzyme kinetics (Bouvier et al. 2021) and why rubisco is one of the slowest evolving enzymes on Earth (Bouvier et al. 2022). Specifically, evolutionary constraints intrinsic to the location, mRNA abundance, and amino acid composition of rubisco (in addition to functional constrains on the protein) may help to explain why it has been so slow to adapt to changing atmospheric conditions, and consequently why it is poorly suited to the O_2_-rich, CO_2_-poor atmosphere of the present day. Furthermore, it is tempting to speculate that endosymbiotic gene transfer to the nuclear genome allowed other plastid-encoded genes to escape some of these evolutionary constraints, facilitated more rapid adaptation of chloroplast proteins, and provided a further advantage for migration of organellar genes to the nuclear genome.

In summary, the molecular adaptation of the land plant chloroplast has been constrained by the location, composition, and expression of its genes. The presence of these evolutionary constraints limits the evolvability of adaptive phenotypes arising from chloroplast-encoded genes and thus motivates strategies to improve photosynthesis in synthetic biology contexts by circumventing these evolutionary barriers to optimization.

## Materials and Methods

### Data Set Curation

The complete set of fully sequenced plastid genomes was downloaded from the NCBI (https://www.ncbi.nlm.nih.gov/) in July 2021. Those genomes that did not contain a full set of canonical plastid-encoded photosystem I, photosystem II, ATPase, NAD(P)H dehydrogenase-like, cytochrome b_6_f complex, ribosomal, and RNA polymerase genes were discarded. All other plastid-encoded genes were not included as they were missing from a large number of angiosperm plastid genomes (supplementary table S2, Supplementary Material online). The full set of genes included in the analysis and their accession numbers are provided in supplementary file 2, Supplementary Material online. The protein sequences encoded by these genes were aligned using MAFFT L-INS-I (Katoh and Standley 2013) and visually checked in AliView (Larsson 2014).

### Phylogenetic Tree Inference

The protein multiple sequence alignments were used to guide codon alignment using Pal2Nal (Suyama et al. 2006) so that there were no inconsistencies between the protein and nucleotide alignments. Nucleotide and protein multiple sequence alignments were trimmed to remove columns containing >90% gaps using trimAl (Capella-Gutiérrez et al. 2009). All 69 trimmed nucleotide multiple sequence alignments were concatenated together and used to identify the best-fitting model of sequence evolution using the inbuilt ModelFinder function in IQ-TREE (Kalyaanamoorthy et al. 2017). In total, 243 models of sequence evolution were tested and the best-fitting model according to the Bayesian information criterion was GTR+F+R7. A maximum likelihood phylogenetic tree was then inferred from the concatenated nucleotide alignment and the best-fit model GTR+F+R7 using IQ-TREE's ultrafast bootstrapping method with 1,000 replicates (Hoang et al. 2018). The resulting tree was then rooted manually in Dendroscope using the Nymphaeales species as the outgroup as they were the most basal-branching clade in the tree (Huson and Scornavacca 2012).

### Substitution Rates and Selection Pressure

To estimate the nonsynonymous and synonymous substitution rates for each individual gene, synonymous and nonsynonymous gene trees were inferred from the trimmed gene nucleotide multiple sequence alignments and the MG94+F3X4 codon substitution model using HYPHY's FitMG94 method (Muse and Gaut 1994; Pond et al. 2004). Here, the topology of the trees was constrained to that identified from the concatenated alignment above. This was done as this is the topology that is most likely to be the true topology for all genes given the uniparental inheritance of the plastid in the absence of sexual recombination—that is, all gene trees should have the same topology. For each nonsynonymous and synonymous gene tree, the total tree length, which is the sum of all branch lengths, was extracted from the HYPHY output and used as a measure of gene-wide nonsynonymous (*d*_N_) and synonymous substitution (*d*_S_) rate. These values were then used to calculate a gene-wide ratio of the rate of nonsynonymous to synonymous substitution (*d*_N_/*d*_S_) which is a measure of selection pressure. Due to the high species sampling and low rate of molecular evolution of the plastid genome, all per-branch estimated values of *d*_S_ and *d*_N_ were less than 0.88 and 0.32, respectively (supplementary table S1, Supplementary Material online). Thus, saturation did not occur on any branch in any tree and therefore could not have influenced the results presented in this study. There were too few indels to facilitate an analysis of indel evolution (supplementary file 3, Supplementary Material online).

### Protein Tolerance to Substitution

Protein tolerance to substitution scores were calculated using the BLOSUM62 matrix (Henikoff and Henikoff 1992). BLOSUM62 was chosen as it is the most widely used substitution matrix in the biological sciences, being used in BLAST (and related sequence search methods) and nearly all protein multiple sequence alignment methods. Here, the odds ratios for all substitutions were back calculated from the log-odds scores provided. Each residue was then given a substitution tolerance by calculating the geometric mean of the odds ratios of its substitution to the remaining 19 common residues. Then, for each of the 69 proteins analyzed, the average residue substitution tolerance was calculated by taking the arithmetic mean of the concatenated protein sequence (composed from all 773 species sequences for that protein).

### Gene Position and GC_3_ Content

Although the plastid genome is a highly conserved structure in the angiosperm lineage, its size, and thus gene position, varies between species. Because all of the genes included in this analysis are in the single-copy region, the average genetic distance of a gene's midpoint to the closest inverted repeat border was used as a measure of gene position relative to the putative origin of replication (the inverted repeat). To do this, the positions of the inverted repeat regions were identified for each species by searching for large-duplicated regions using BLASTN (Altschul et al. 1990; Camacho et al. 2009). Then, the distance between the gene's midpoint (obtained from the GenBank file for each species’ plastid genome) and the closest inverted repeat border was calculated. An average distance was then determined and this average for each gene was used in the analysis of gene position. Positional effects were also analyzed at the level of operons. To do this, operon maps were obtained from Shahar et al. (2019) for six diverse species in our data set. The operon's distance to the closest inverted repeat border was determined by averaging the distances of its encoded genes.

The average GC content of the third codon position (GC_3_) for all 773 species was calculated for each gene. The GC_3_ excluded methionine and tryptophan codons as these amino acids are encoded by a single codon and thus have no flexibility in base identity at the third position.

### mRNA Abundance and Protein Abundance

mRNA abundance data were obtained from Forsythe et al. (2022) and values were available for all 69 genes analyzed in this study. The whole-organism integrated (3702-WHOLE_ORGANISM-integrated.txt) protein abundance estimates for *Arabidopsis thaliana* were obtained from the Protein Abundance Database (PAXdb) (Wang et al. 2012). Abundance data were available for 56/69 genes included in this analysis.

### Transcript Biosynthetic Cost and Translational Efficiency

The strength of selection acting to reduce transcript cost, increase translational efficiency, and the trade-off between these two forces was inferred for each species using CodonMuSe (Seward and Kelly 2018). The tRNA copy numbers required by CodonMuSe were inferred for each species by inputting the species plastid genome to tRNAscan-SE 2.0 (Chan et al. 2021).

### Statistical Analysis and Modeling

All statistical analysis and linear regressions were calculated in R. Identification of the best subset of variables for multiple linear regressions was determined using the olsrr package. Relative importance metrics were generated using the reliampo package (Groemping 2006). Random forests were using the randomForest package (Svetnik et al. 2003).

## Supplementary Material

evad101_Supplementary_Data

## Data Availability

All data used in this study are provided in the Supplementary Material online, and all accession numbers are provided.
