# Supplementary material for: The Evolutionary Constraints on Angiosperm Chloroplast Adaptation
Source: Genome Biol Evol. 2023 Jun 3;15(6):evad101. doi: 10.1093/gbe/evad101 (PMC10279810; doi:10.1093/gbe/evad101)
Supplement: evad101_Supplementary_Data [file evad101_supplementary_data.zip › Supplemental Figures.docx]

***Supplementary Figure S1***

**
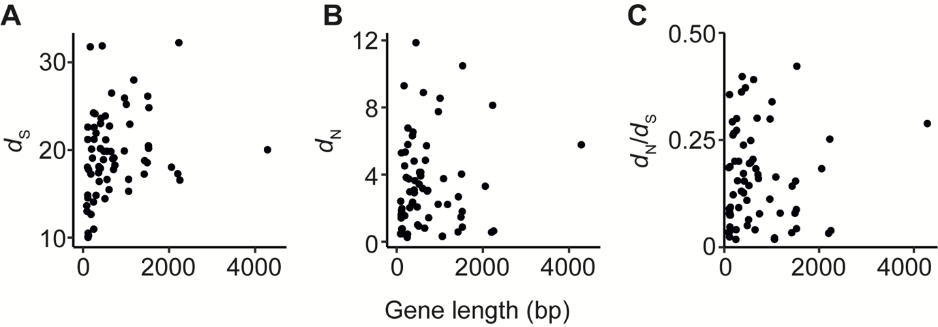
**

**Supplementary Figure S1.** Relationships between the rates of molecular evolution and sequence length for 69 plastid-encoded genes. Scatter plots of the rate of synonymous substitution (*d*_S_) (**A**), non-synonymous substitution (*d*_N_) (**B**) and the ratio of non-synonymous to synonymous substitution (*d*_N_/*d*_S_) (**C**) versus the average gene length given in base pairs (bp). *d*_S_ has the units of number of synonymous changes per synonymous sequence site and *d*_N_ has the units of the number of non-synonymous changes per non-synonymous sequence site.

***Supplementary Figure S2***

***
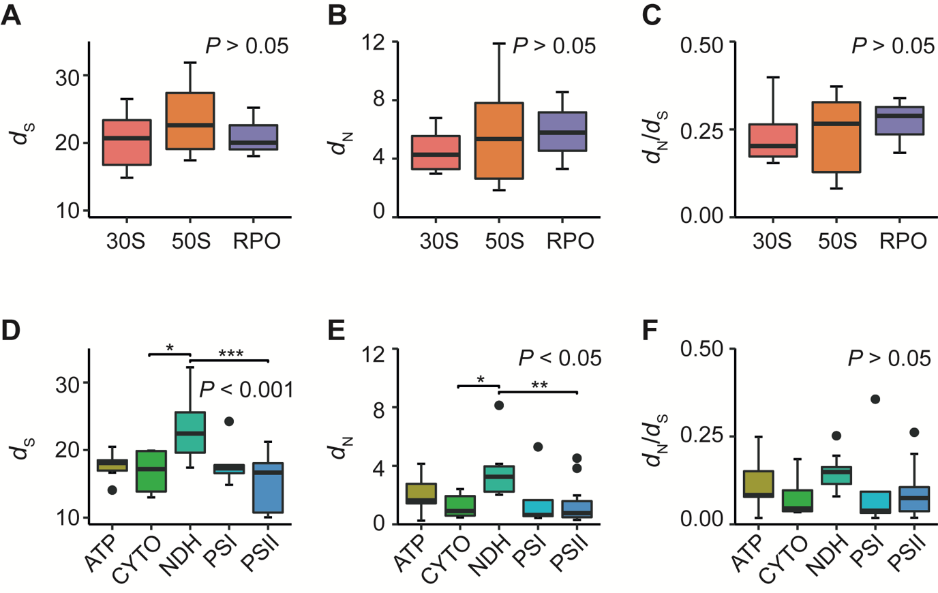
***

**Supplementary Figure S2.** Exploring the differences in rates of molecular evolution and functional constraint between the multiprotein complexes encoded by the plastid. The difference in the rate of synonymous substitution, rate of non-synonymous substitution and functional constraint between the major multiprotein complexes of the information processing category (**A-C**) and energy production category (**D-F**), respectively. Groups include the large ribosomal subunit (50S, n = 7), small ribosomal subunit (30S, n= 10), RNA polymerase (RPO, n = 3), photosystem I (PSI, n = 5), photosystem II (PSII, n = 15), cytochrome b_6_f complex (CYTO, n = 6), ATP-synthase (ATP, n = 6) and NADPH dehydrogenase-like complex (NDH, n = 10). Statistical significance was assessed using one-way ANOVAs and their associated *P*-values are displayed. Where appropriate, asterisks indicate the *P*-value significance levels of *post-hoc* Tukey tests.

***Supplementary Figure S3***

**
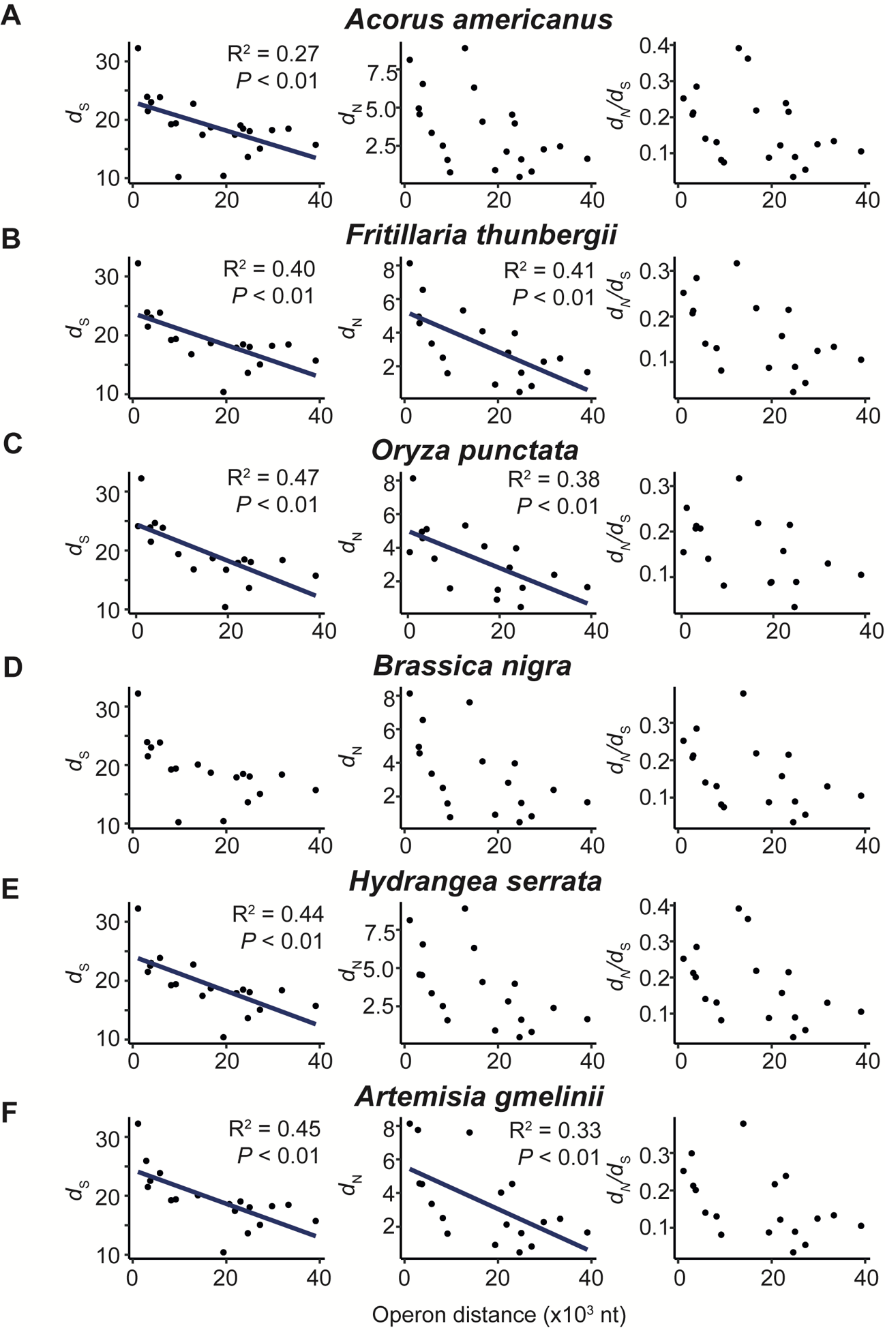
Supplementary Figure S3.** The relationship between the distance of an operon to the closest inverted repeat border and the rate of molecular evolution and strength of purifying selection of its encoded genes for six species from across the tree analysed in this study. These species included three monocots and three eudicots, all from different orders. The rate of synonymous substitution, non-synonymous substitution and strength of purifying selection of an operon is calculated from the average rate of molecular evolution of its encoded genes that were included in this analysis. Scatter plots showing the average distance of each operon to the closest inverted repeat border measured in nucleotides (nt) versus the rate of synonymous substitution (left), non-synonymous substitution (middle) and ratio of non-synonymous substitution to synonymous substitution (right) for *Acorus americanus* (**A**), *Fritillaria thunbergia* (**B**), *Oryza punctata* (**C**), *Brassica nigra* (**D**), *Hydrangea serrata* (**E**) and *Artemisia gmelinii* (**F**). Linear models are shown in blue with their associated R^2^ and *P*-values.

***Supplementary Figure S4***


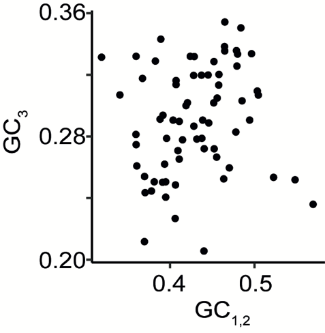


**Supplementary Figure S4.** The relationship between the GC content of the first and second codon positions (GC_1,2_) versus the GC content of the third codon position (GC_3_) for 69 plastid-encoded protein coding genes.

***Supplementary Figure S5***


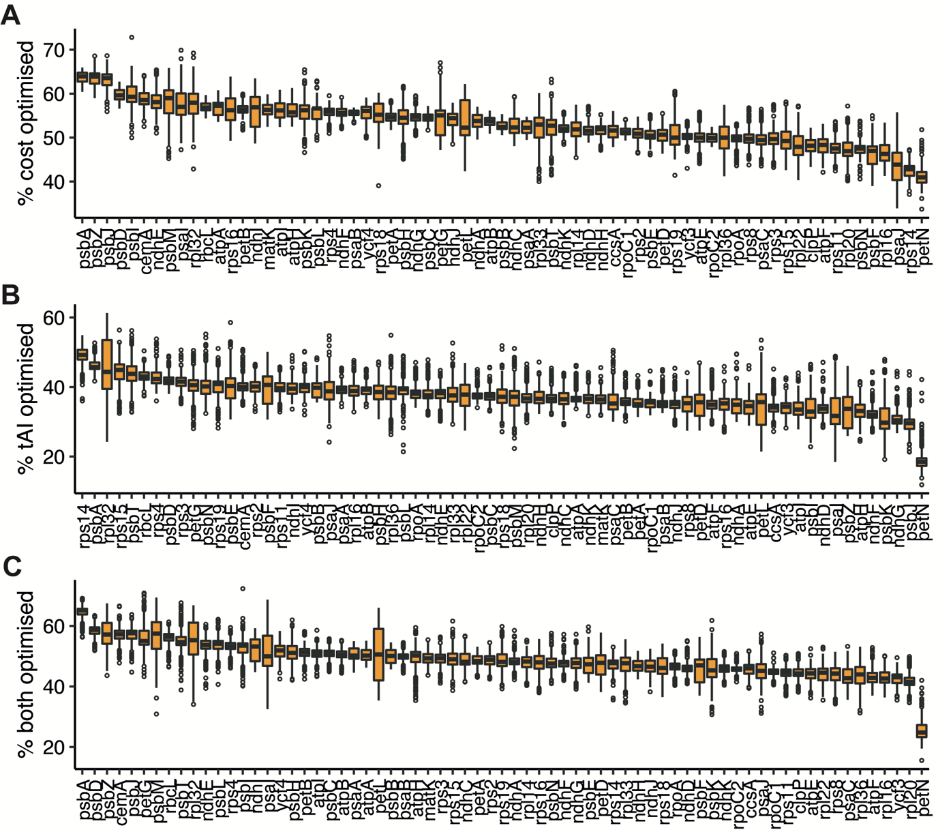


**Supplementary Figure S5.** Extent of gene optimisation for transcript biosynthetic cost and translational efficiency for 69 plastid-encoded genes. For each gene, CodonMuse was used to calculate percent optimisation values for transcript biosynthetic cost (**A**), translational efficiency (**B**) and the trade-off between these two evolutionary forces (**C**) for 773 species which are represented as boxplots. Genes are arranged with decreasing median values from left to right.

***Supplementary Figure S6***

***
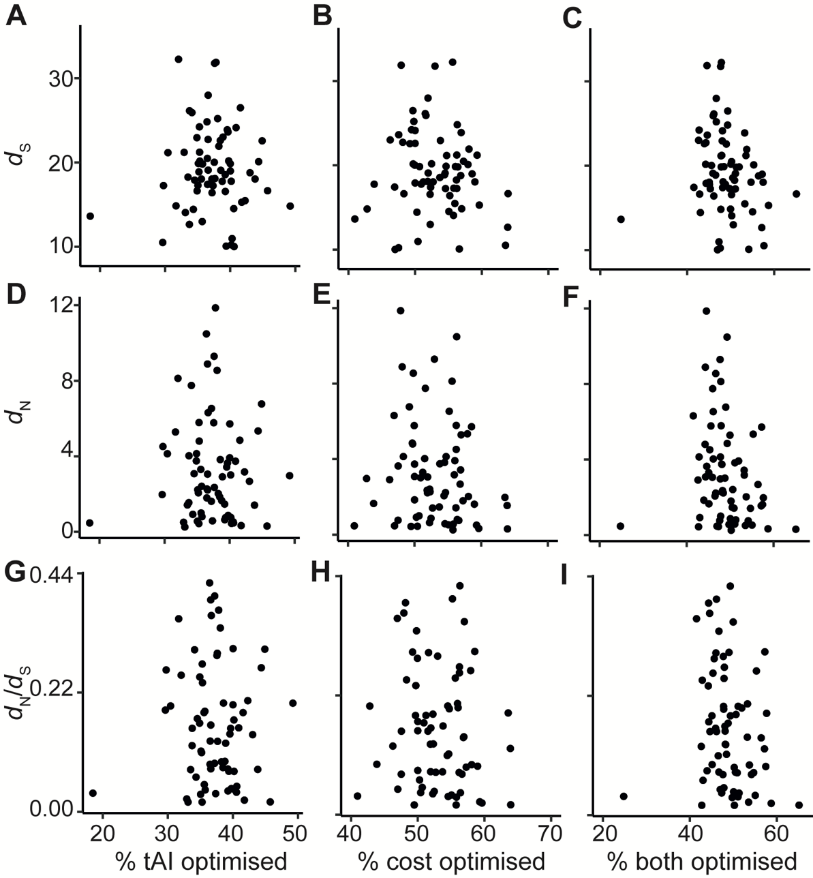
***

**Supplementary Figure S6.** The relationship between the extent of optimisation of transcript cost and translational efficiency with the rate of molecular evolution. Optimisation values were calculated using CodonMuSe and are given as a percentage, where 100% is the most optimised nucleotide sequence for a given protein sequence. The translational efficiency optimisation score is based on the tRNA adaptation index (tAI) and the cost optimisation scores are based on codon nitrogen cost. Relationships between the median of gene optimisation for translational efficiency, transcript cost and the trade-off with the rate of synonymous substitution (**A-C**), the rate of non-synonymous substitution (**D-F**) and the strength of selection pressure (**G-I**), respectively.

***Supplementary Figure S7***

**
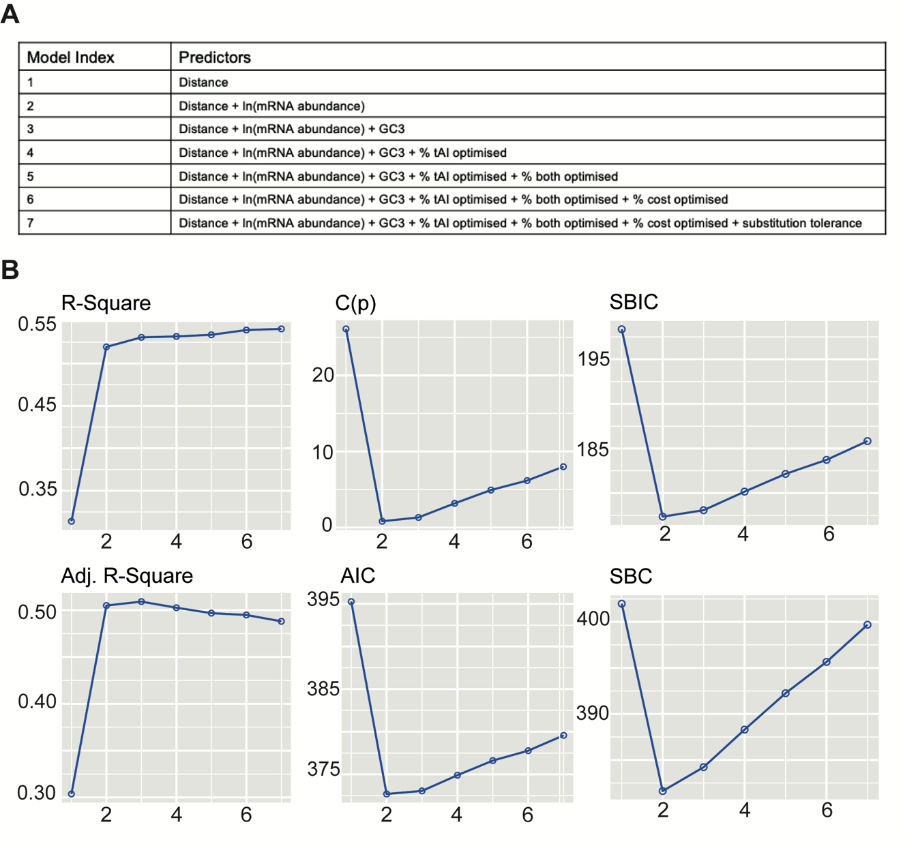
**

**Supplementary Figure S7.** Variable selection for the best-fitting linear model explaining the maximum variation in the rate of synonymous substitution. **A**) Table showing the subset of variables used for the different models tested (indexed 1-8). Variables include average gene distance to the inverted repeat, Distance; the natural logarithm of transcript abundance, ln(mRNA abundance); GC content of the wobble position, GC_3_, mean percent optimisation for translational efficiency, % tAI optimised; mean percent optimisation for transcript cost, % cost optimised; mean trade-off for optimisation of both translational efficiency and transcript cost, % both optimised and protein tolerance to substitution, substitution tolerance. **B**) Various objective criteria values for the models described in A. R-Square, R-squared of the model; Adj. R-Square, R-squared after adjusting for the number of parameters; C(p), Mallow’s Cp; AIC, Akaike information criterion; SBIC, Sawa’s Bayesian information criteria; SBC, Schwarz Bayesian information criteria.

**Supplementary Figure S8**

**
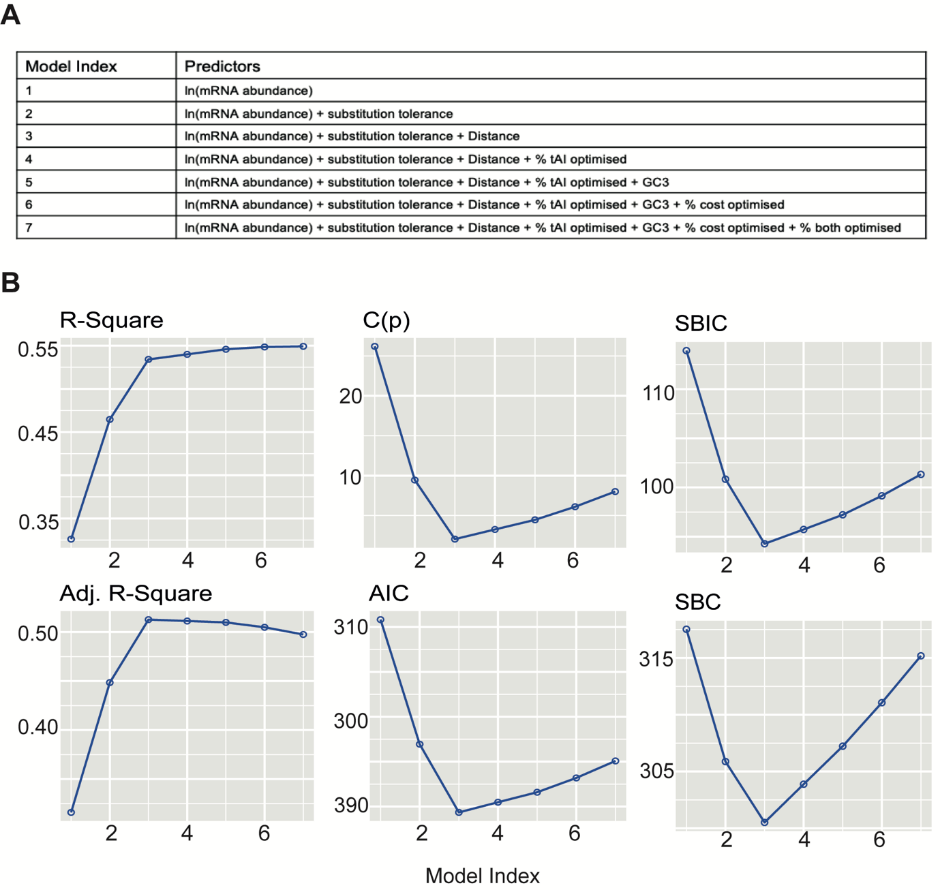
**

**Supplementary Figure S8.** Variable selection for the best-fitting linear model explaining the maximum variation in the rate of non-synonymous substitution. **A**) Table containing the subset of predictors for each model tested. **B**) Various object criterion values for the models described in A.

***Supplementary Figure S9***

***
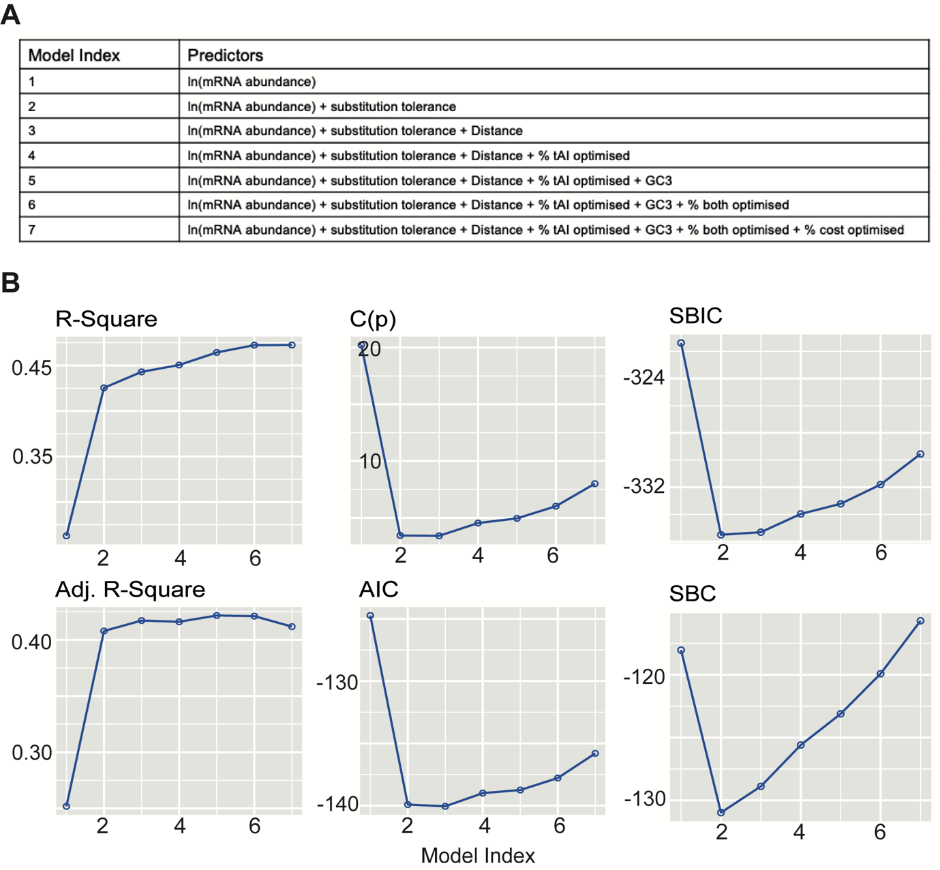
***

**Supplementary Figure S9**. Variable selection for the best-fitting linear model explaining the maximum variation in the ratio of the rate of non-synonymous substitution to the rate of synonymous substitution. **A**) Table containing the subset of predictors for each model tested. **B**) Various object criterion values for the models described in A.
