# Supplementary material for: The Evolutionary Constraints on Angiosperm Chloroplast Adaptation
Source: Genome Biol Evol. 2023 Jun 3;15(6):evad101. doi: 10.1093/gbe/evad101 (PMC10279810; doi:10.1093/gbe/evad101)
Supplement: evad101_Supplementary_Data [file evad101_supplementary_data.zip › Supplementary File 1.docx]

***Supplementary File 1***

Random forest regressions

Random forest regressions were used to see how much variance in the rate of synonymous mutation, rate of non-synonymous mutation and the strength of purifying selection could be explained by the variables explored in this study. The input for the random forest regressions included gene distance to the inverted repeat, protein substitution tolerance (see methods), gene GC_3_ content, mRNA abundance, protein abundance, % optimised for transcript biosynthetic cost, % optimised for translational efficiency and % optimised for the trade-off between transcript cost and translational efficiency.

*Rate of synonymous mutation*

The percentage of variance in the rate of synonymous mutation explained by the random forest model using all input variables was 41% (mean squared of residuals = 14.1). This is less than the variance explained by the best multiple linear regression model (52%, see results) which contained only 2 covariates, gene distance to the inverted repeat border and mRNA abundance. However, in agreement with our linear models, the random forest regression identified these two co-variates as the most important variables (Table 1, Figure 1).


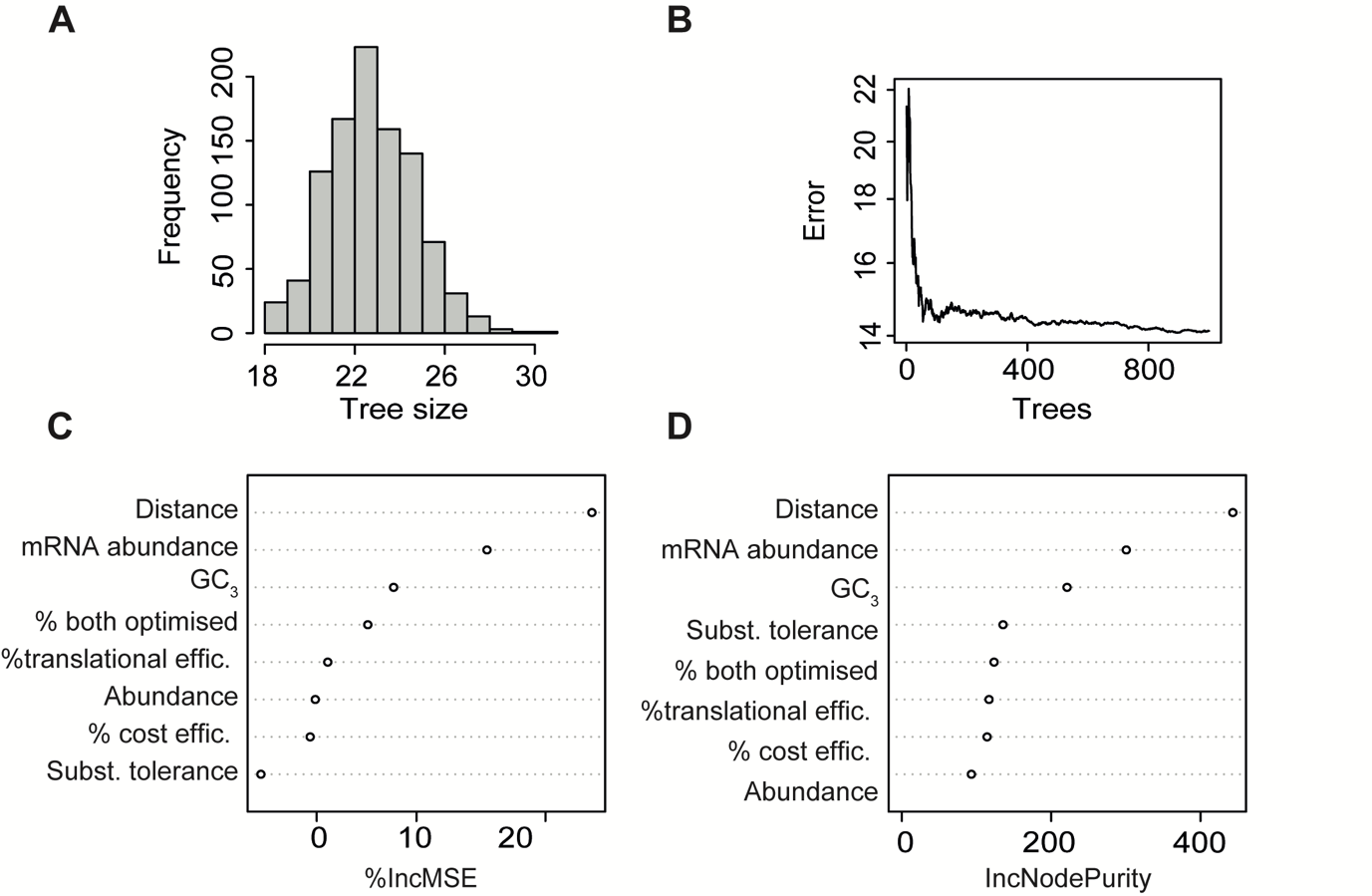


**Figure 1.** Random forest regression for the rate of synonymous mutation. **A)** Histogram of tree size for the 1000 trees generated. **B)** Error rate versus the number of trees built. **C)** The percentage increase in mean squared error (%IncMSE) for all variables included in the regression ordered in decreasing value from top to bottom. **D)** The increase in node purity (IncNodePurity) in decreasing value from top to bottom.

**Table 1.** Variable importance metrics for random forest regression of the rate of synonymous mutation.

*Rate of non-synonymous mutation*

The percentage of variance in the rate of non-synonymous mutation explained by the random forest model using all input variables was 31% (mean squared of residuals = 4.94). As for the rate of synonymous mutation, this was also less than the variance explained by the best multiple linear regression model (53%, see results) which contained 3 covariates, gene distance to the inverted repeat, protein substitution tolerance and mRNA abundance. However, in agreement with our linear models, the random forest regression identified these three co-variates as the most important variables (Table 2, Figure 2).


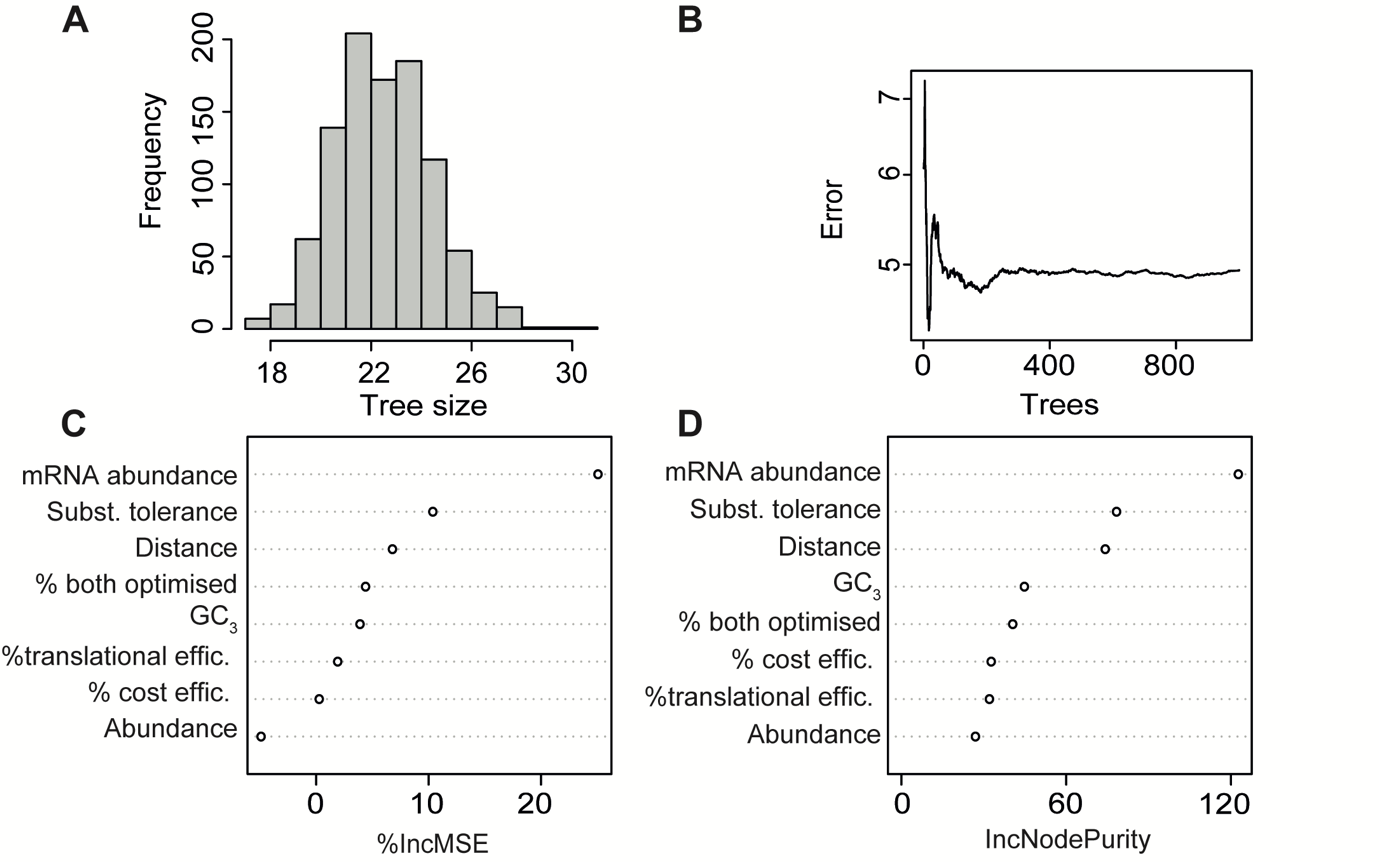


**Figure 2.** Random forest regression for the rate of non-synonymous mutation. **A)** Histogram of tree size for the 1000 trees generated. **B)** Error rate versus the number of trees built. **C)** The percentage increase in mean squared error (%IncMSE) for all variables included in the regression ordered in decreasing value from top to bottom. **D)** The percentage node purity (IncNodePurity) in decreasing value from top to bottom.

**Table 2.** Variable importance metrics for random forest regression of the rate of non-synonymous mutation.

*Strength of purifying selection*

The percentage of variance in the strength of purifying selection (ratio of the rate of non-synonymous mutation to synonymous mutation) explained by the random forest model using all input variables was 26% (mean squared of residuals = 0.009). As for both the rate of non-synonymous mutation and synonymous mutation, this was also less than the variance explained by the best multiple linear regression model (43%, see results) which contained 2 covariates, protein substitution tolerance and mRNA abundance. However, in agreement with our linear models, the random forest regression identified these two co-variates as the most important variables (Table 3, Figure 3).


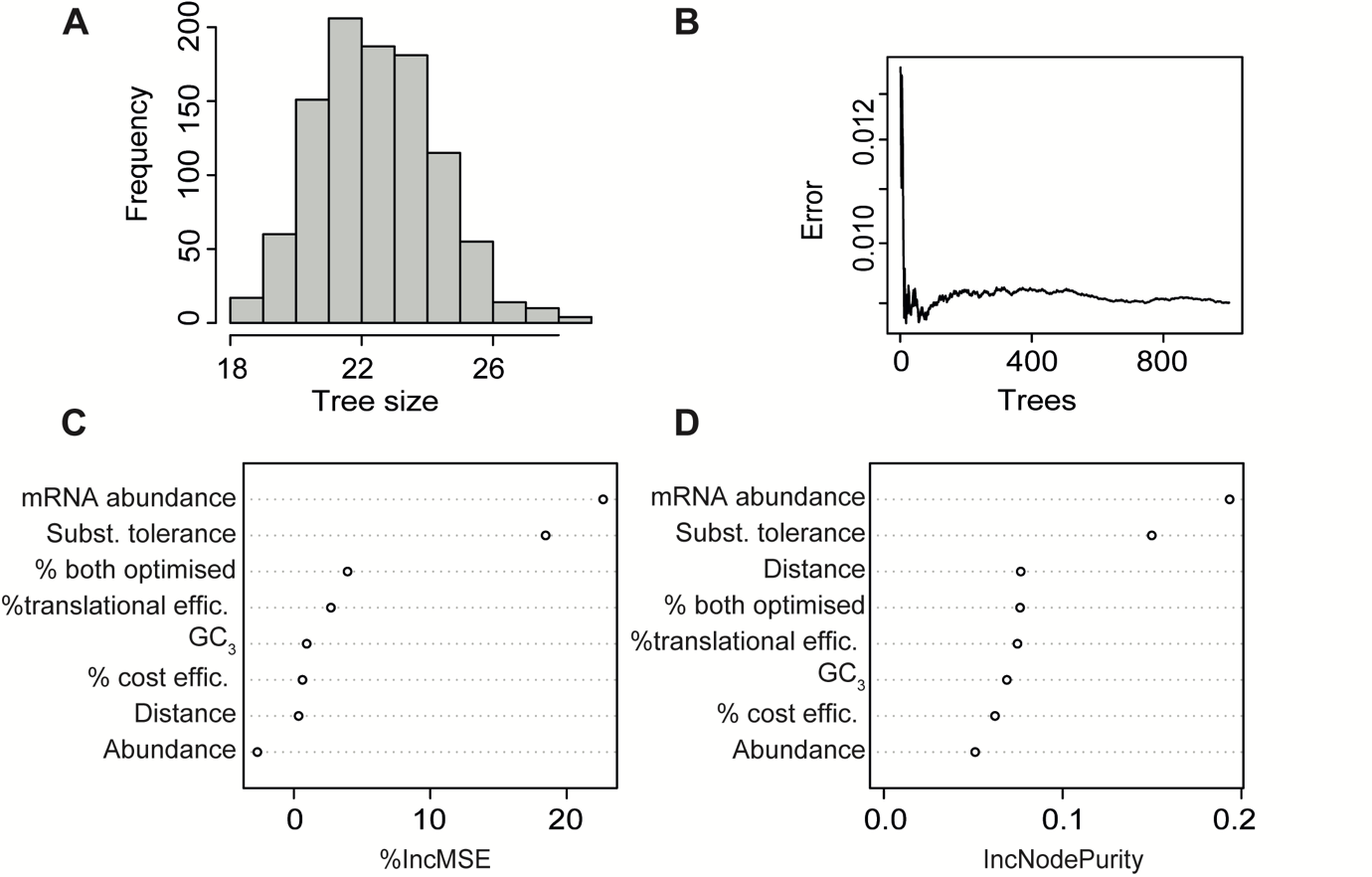


**Figure 2.** Random forest regression for the strength of purifying selection. **A)** Histogram of tree size for the 1000 trees generated. **B)** Error rate versus the number of trees built. **C)** The percentage increase in mean squared error (%IncMSE) for all variables included in the regression ordered in decreasing value from top to bottom. **D)** The percentage node purity (IncNodePurity) in decreasing value from top to bottom.

**Table 2.** Variable importance metrics for random forest regression of the strength of purifying selection.
